# Validation of a real-time polymerase chain reaction for the detection and quantification of the nucleic acid of *Histoplasma* from equine clinical samples

**DOI:** 10.1128/spectrum.03100-23

**Published:** 2024-02-27

**Authors:** Lewis W. S. Fisher, Abdou Ceesay, Demba Jallow, Sophie F. Hawkes, Alicia Showering, Yaghouba Kane, Amadou Doumbia, Andrew P. Stringer, Claire E. Scantlebury

**Affiliations:** 1 The Faculty of Health and Life Sciences, Institute of Infection, Veterinary and Ecological Sciences, Department of Livestock and One Health, The University of Liverpool, Liverpool, United Kingdom; 2 Department of Livestock Services, Ministry of Agriculture, Abuko, Gambia; 3 Department of Public Health and Environment, Ecole Inter Etats des Sciences et Medecine Veterinaires de Dakar, Dakar, Senegal; 4 SPANA, Bamako, Mali; 5 Department of Population Health and Pathobiology, College of Veterinary Medicine, North Carolina State University, Raleigh, North Carolina, USA; Uniwersytet Medyczny w Bialymstoku, Bialystok, Poland

**Keywords:** qPCR *Histoplasma*, detecting *Histoplasma* equine samples, equine clinical samples, *Histoplasma* and West Africa

## Abstract

*Histoplasma capsulatum* var*. farciminosum* (HCF) is a dimorphic fungus that causes epizootic lymphangitis in equids. Current diagnostic approaches, including culture, microscopy, and clinical presentation, lack speed, sensitivity, and specificity when diagnosing clinical cases. In this study, equine blood and pus samples on Whatman FTA cards from Senegal (*n* = 3), The Gambia (*n* = 19), Ethiopia (*n* = 16), and Mali (*n* = 13) were tested using a real-time PCR (qPCR) protocol. The assay was optimized and tested for its suitability to detect and quantify HCF in blood and pus loaded onto Whatman FTA cards at sampling. Whatman FTA cards were tested for their suitability for use with qPCR and were found to recover DNA more efficiently than from direct extraction. Using TaqMan fluorescent probes and specific primers, the assay demonstrated 100% analytical specificity when detecting multiple strains of *Histoplasma* and no false positives with off-target organisms. The assay’s diagnostic performance was measured against an existing nested internal transcribed spacer PCR protocol using a receiver operating characteristic curve. The test was found to have a diagnostic specificity and sensitivity of 100% and 71.4%, respectively, when analyzing pus samples using a cycle threshold (Ct) cutoff determined by Youden’s index (27.75). Blood sample cutoff Ct value was proposed at 34.55. Further optimization is required to improve the performance of the protocol when applied to blood samples. This study has, for the first time, demonstrated the ability to detect and quantify the DNA of *Histoplasma* spp. in equine blood and pus samples with a high degree of accuracy, providing a platform to further investigate the pathogenesis and epidemiology of this disease.

**IMPORTANCE:**

Histoplasmosis is a neglected yet major cause of morbidity and mortality in both equids and people in resource-scarce settings. One of the major hindrances to the control of histoplasmosis is a lack of readily available diagnostic tests. Tests are needed to support clinical decision-making and to be applied in population-based research to further understand this disease *in situ*. This paper reports, for the first time, the validation and application of a qPCR to detect *Histoplasma* directly from equine clinical samples, bypassing the need to culture this notoriously difficult organism. We report and comment on the performance of the qPCR in comparison with our previously developed nested PCR.

## INTRODUCTION

Epizootic lymphangitis (EZL) is an infectious disease caused by the dimorphic fungal pathogen *Histoplasma capsulatum* var. *farciminosum* (HCF). EZL predominantly affects equids, such as donkeys, mules, and horses. Working equids are relied upon in low-income countries for farming and commercial activities such as transporting people, goods, crops, market products, and removal of household waste in both rural and urban settings, making them an important source of fiscal and social capital (1). The morbidity or mortality of a horse, donkey, or mule can have a devastating impact on the owner’s livelihood (2). In Ethiopia, the prevalence of EZL is estimated to be between 0% and 39% depending on region, with an average prevalence of 18.8% (3).The regional differences in prevalence are thought to be attributed to temperature, humidity, and population density of equids (3, 4). Historic surveillance data have suggested that EZL only occurred in Ethiopia, Senegal, and South Africa (5). However, reports of clinical cases in countries such as The Gambia and Chad suggest that the disease may be more widespread in Africa (6).

Four clinical presentations of EZL are described in equids, such as cutaneous, ophthalmic, respiratory, and asymptomatic forms, but mixed forms also occur (7, 8). Clinical lesions typically comprise pyogranulomatous subcutaneous nodules that may be located on any part of the body but are most often present on the limbs. Nodules spread via lymphatic vessels which can develop a corded appearance, with subsequent lymphangitis. Advanced EZL is severely debilitating with lameness resulting from lymphangitis and associated swelling of the joints and limbs. Owners have limited management options due to financial restrictions, lack of available anti-fungal treatment, and limited access to veterinary care. In Ethiopia and The Gambia, treatments are only available through non-governmental organization clinics that use a combined protocol of imported oral potassium iodide along with topical 4% iodine tincture. This form of treatment is expensive, labor intensive, and prolonged and is not effective for moderate to advanced cases of EZL (9).

Currently, animal health professionals presumptively diagnose EZL based on clinical presentation, light microscopy, and, rarely, microbial culture. However, other diseases, including sporotrichosis, the cutaneous presentation of glanders, strangles, and ulcerative lymphangitis (7), can have similar presentations, and relying on clinical signs alone could lead to misdiagnosis. Light microscopy relies on visualization of ovoid yeast cells within histological sections and pus exudates; however, misdiagnosis with other fungal infections is possible (8, 10). The gold standard for the diagnosis of histoplasmosis in humans relies on the culture of *Histoplasma capsulatum* var. *capsulatum* (HCC). However, culturing *Histoplasma* is time consuming, requiring 2–12 weeks for confirmation (11, 12).

Molecular methodologies to diagnose clinical cases of EZL have received little attention in the past; in contrast, there have been many reports of molecular assays to detect human histoplasmosis (HCC). Our laboratory group previously (8) reported the direct detection of HCF in equine blood and pus samples captured on Whatman FTA cards targeting the internal transcribed spacer (ITS) region using a nested PCR method. Real-time PCR has been used to identify *Histoplasma capsulatum* within an array of human clinical samples (13–15) with a high degree of sensitivity, one study detecting as few as 100 DNA copies/µL (14). Protocols have been reported for qPCR detection of other fungal pathogens such as *Blastomyces dermatitidis*, *Cryptococcus neoformans*, *Cryptococcus gattii*, *Coccidioides immitis,* and *Coccidioides posadasii* (15–17). The use of qPCR as a diagnostic method can vastly improve the speed, reliability, and analytical sensitivity compared with other established methods of diagnosis such as conventional qualitative PCR.

There are both structural and socio-economic challenges in establishing molecular methodologies to detect endemic histoplasmosis in low-resource settings where diagnosis of equine EZL is already constrained by a lack of laboratory consumables and equipment, intermittent power supply, and unreliable communication networks. These constraints need to be addressed to support the development and adoption of accurate, rapid, and sensitive diagnostic methodologies to support animal health professions and diagnostic laboratories in Africa and globally. This will aid in identifying clinical cases as well as increasing knowledge on the epidemiology, transmission, and maintenance of EZL. Furthermore, improving diagnostic facilities will reduce unwarranted use of antimicrobials and allow more targeted treatment of disease, thus reducing antimicrobial resistance.

This study aimed to validate the use of a qPCR protocol reported by Simon et al. (13) in equine pus and blood samples to examine its use in providing presence/absence data and for quantification of DNA extracted from banked equine clinical samples stored on Whatman FTA cards.

## MATERIALS AND METHODS

### Clinical samples

**Fig 1:**
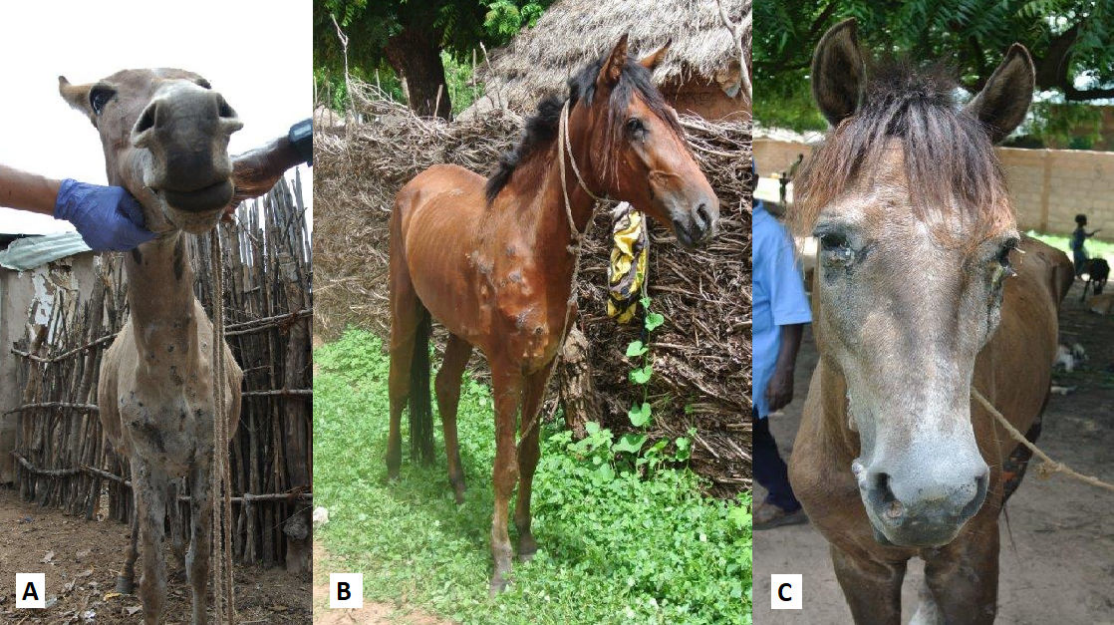
Varieties of clinical presentation of epizootic lymphangitis among equine cases in The Gambia. (**A**) Donkey with extensive severe cutaneous EZL, with cording on both forelimbs, involvement of axillary lymph nodes and extension to the chest and neck.(B) A young horse with moderate cutaneous EZL affecting the right forelimb and chest region. Clusters of variably ruptured pyogranulomatous cutaneous lesions are visible. (**C**) A severe case with respiratory, cutaneous, and ocular signs. There are ulcerative plaques on the muzzle and within the nasal mucosa, pyogranulomatous lesions around each eye with purulent discharge, and skin excoriation.

The data set comprised clinical samples from four different African countries, such as The Gambia, Senegal, Mali, and Ethiopia. Blood and pus samples were collected from animals suspected to have EZL based on clinical signs and/or microscopic examination of pus exudates. Prior to collecting pus samples, each animal was sedated using a combination of butorphanol and detomidine to allow safe and pain-free collection of pus; following this, all EZL cases were treated with a combination of potassium iodide and tincture of iodine. No animal was euthanized for the purposes of this study. Cases were selected on the basis of clinical signs suggestive of infection with HCF (Fig. 1), provided that they presented unruptured nodules allowing sampling (8).

Confirmatory diagnosis was examined using a nested-PCR test (8) (Table S1). A grading system, used routinely by the Society for the Protection of Animals Abroad (SPANA) clinics in Ethiopia, was adopted to characterize the clinical appearance and distribution of lesions among the cases. This allowed us to group cases as mild, moderate, and severe (9), details of which are provided (Table S2). Cases were excluded if they did not have any lesions suggestive of HCF, if the animal was fractious and not amenable to sampling, or if the owners did not wish to contribute samples for the study. The University of Liverpool Research Ethics Committee, The Department of Livestock Services, The Gambia, and The Gambia Horse and Donkey Trust approved this study. Owners were invited to provide voluntary informed consent to join the study, and written consent was obtained through translators.

Twenty blood samples (17 from horses and 3 from donkeys) and 13 pus samples (10 horses and 3 donkeys) were collected from 8 sites across different areas of the central and upper river regions of The Gambia. Nineteen blood samples and 13 pus samples were eligible to be used in this study. All cases attended clinics run by the Department of Livestock Services and The Gambia Horse and Donkey Trust; basic clinical examination and hematological values were recorded (Tables S2 and S3). Any cases presenting with EZL were treated by infusion of tincture of iodine and oral potassium iodide [as per reference (8)]. Two blood samples and three pus samples were collected from three horses suspected of EZL infection in Senegal based on clinical signs. Specimen collection was facilitated by the veterinarians at Ecole Inter Etats des Sciences et Médecine Vétérinaires in Dakar. One blood sample and three pus samples were eligible to be used in this study (Table S1). Thirteen blood samples from horses in Mali were collected by SPANA clinic staff in Mali based on clinical appearance of EZL, and 16 blood samples from horses originating from various sites in Ethiopia were obtained from a previous data set (18). The samples from Ethiopia were selected from a large cross-sectional study and expected to be negative for EZL based on the absence of clinical signs (Table S2) (18). These blood specimens were included by way of exploring the test performance on samples from clinically negative animals. All clinical samples were collected according to methods described in reference (8) and stored on Whatman FTA cards (GE Healthcare) at ambient temperature, then 4°C until the DNA extractions were performed.

Genomic DNA was extracted from the Whatman FTA cards using a phenol-chloroform extraction method as described in reference (8); this included a bead beating step with the incorporation of 0.5 g acid-washed glass beads (diameter, 425–600 µm) to effectively disrupt the FTA card matrix. The phenol-chloroform extraction method was followed as described in reference (8), with the exception that if samples were clearly discolored or cloudy, the chloroform step was repeated to further purify the samples. Following the separation of the aqueous phase, 1 mL of 100% ethanol 5 µL of 200 mM sodium acetate trihydrate (VWR, catalog number: 27648.294) was added to the samples followed by incubation at −20°C for 24 h. Subsequently, the samples were centrifuged at 13,000 × *g* for 10 min, and the supernatant was discarded. The DNA pellet was thoroughly air dried and then rehydrated in 50 µL nuclease-free water.

### Primers and probe for qPCR

Published primers and probes (13) were used to develop the procedure for the detection of *Histoplasma capsulatum* var. *farciminosum*. The probe was HcITS188P 5′-6FAM-AGAGCGATAATAATCC-MGB-3′, a custom oligonucleotide probe labeled with 6-carboxyfluorescein (6FAM) at the 5′ end and, at the 3′ end, a non-fluorescent quencher with a minor groove binder (MGB) (Fisher Scientific, custom probe). The primers were custom oligonucleotides HcITS167F 5′-AACGATTGGCGTCTGAGCAT-3′ and HcITS229R 5 ′-GAGATCCGTTGTTGAAAGTTTTGA-3′ (Eurofins, custom oligonucleotides). The analytical specificity of the primers was assessed by using the Primer BLAST tool (19), while the specificity of the oligonucleotide probe was analyzed using BLASTn (20).

### Plasmid DNA standards

#### Conventional PCR

Standards were created by first running a conventional PCR with the forward and reverse primers described previously to create amplicons suitable for TA (thymine and adenine) cloning. MyTaq Red Mix was used according to the manufacturer’s instructions. Fifty-microliter reactions were made using 25 µL of 2 × MyTaq Red Mix, 4.5 µL 10 µM forward primer (900 nM), 4.5 µL 10 µM reverse primer (900 nM), and 100 ng of gDNA from HCF (Westerdijk Fungal Biodiversity Institute, Strain: CBS 176.57) and made up to 50 µL using nuclease-free water (Severn Biotech Ltd). Negative controls were prepared using equal volumes of nuclease-free water in place of the gDNA. The PCR assay was run using the Master Cycler Pro S (Eppendorf) with the program 1 × hot start 95°C 3 min; 30 × 95°C 15 s, 52.8°C 15 s, and 72°C 30 s; and 1 × final extension 72°C 1 min. PCR amplicons were visualized using a UV transilluminator on 3% agarose (Bioline, Catalog number: BIO-41026) using 1× TAE buffer and stained with 8 µL of Midori Green (Nippon Genetics) per 100 mL of agarose. PCR products were run alongside a 25-bp HyperLadder V (Bioline) DNA ladder to confirm the size of the fragment was 63 base pairs in length.

#### TA cloning protocol

The PCR amplicons were purified using an Isolate II PCR and Gel PCR Clean-Up Kit (Bioline), and the concentration of DNA was measured using a Denovix DS-11 FX+ spectrophotometer (Cambridge Bioscience). The purified amplicons were inserted into a plasmid vector and cloned into *Escherichia coli* cells using a pGEM T-easy Vector (Promega) cloning kit. The ligation reaction was incubated for 1 h at room temperature. Following ligation, the plasmid was used to transform competent *E. coli* JM109 cells (Promega) using a heat shock with 50 µL of competent cells and 2 µL ligated plasmid. All steps of the procedure were followed, except where LB broth (Sigma) was used in place of SOC medium. Subsequently, 50 µL of the transformed cells was added to 950 µL of LB broth and the inoculum incubated at 37°C for 1.5 h on an orbital shaker (150 rpm). Then, 100 µL of the inoculum was spread onto LB/ampicillin/IPTG/X-GAL plates and incubated at 37°C overnight. Following incubation, white colonies were picked and sub-cultured onto LB/ampicillin/IPTG/X-GAL plates and incubated for a further 18–24 h. When pure cultures were obtained, a single colony was inoculated into 10 mL LB broth and incubated at 37°C overnight. Once incubations had finished, a plasmid DNA extraction was performed using the Isolate II Plasmid Mini Kit (Bioline) following the low copy number protocol. All steps of the procedure were followed exactly, except for the final elution step where nuclease-free water was utilized.

### Real-time PCR

Plasmid DNA concentration was measured using a Qubit dsDNA HS (high sensitivity) Assay Kit (Life Technologies) and diluted to between 10 pg/µL and 100 ng/µL to be within the recommended range. The copy number of the plasmid was determined using the ThermoFisher DNA copy number and dilution calculator (21). The size of the plasmid DNA fragment (3,063 bp) containing the ITS insert was input into the DNA copy number calculator along with the concentration of the DNA within the extracted sample.

The plasmid standards were diluted to give a final concentration of 10^8^ copies per 20 µL qPCR reaction. The 10^8^ standards were used to create a 1:10 serial dilutions ranging from 10^8^ to 100 copies per reaction.

Real-time PCR assays were run on the Step One Plus Real-Time PCR System (Applied Biosystems). Using the primers and TaqMan fluorescent probe described above, the assay was optimized to use a final reaction volume of 20 µL with 80 nM forward primer, 480 nM reverse primer, and 240 nM TaqMan MGB probe. The assays were run with standards created to have concentrations ranging from 10^8^ copies to 100 copies per 20 µL reaction volume. All the assays were prepared using 2 × TaqMan Gene Expression Master Mix (Applied Biosystems, Catalog number: 4369016). Reactions containing clinical samples (as described in Table S2) were tested using 2 µL of clinical samples extracted using the DNA extraction method outlined above. Samples were run with a cycle program of 1 × 2 min 50°C, 1 × 10 min 95°C, and 40 × 15 s 95°C followed by 1 min 60°C according to the TaqMan Gene Expression Master Mix protocol.

**Fig 2:**
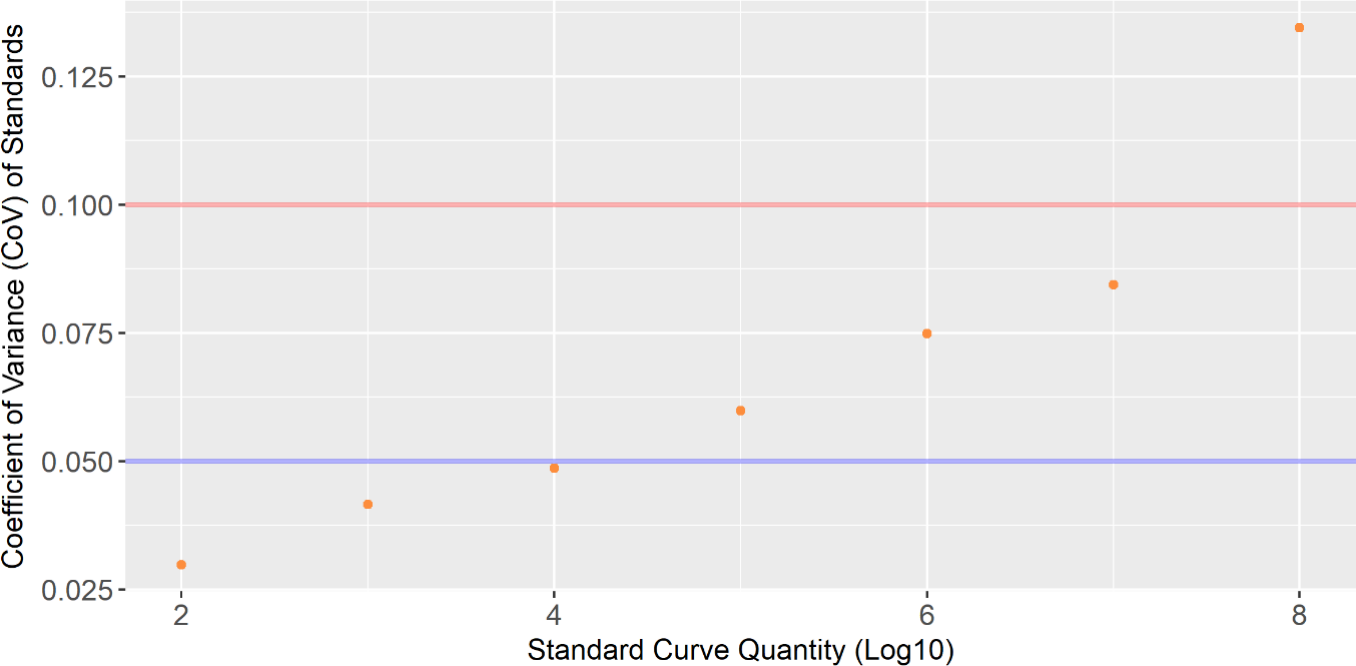
The coefficient of variance plotted against the log10 copy number of a series of standard curves. The blue line depicts the variation between assays is 5%, while the red line indicates 10% variations between assays.

The analytical sensitivity of the assays was assessed using a series of standard curves. The approach for testing the detection limit of the assay was to take a series of five (duplicate) standard curves ranging from 10^8^ to 100 and determine the coefficient of variance (CoV). In total, 10 Ct values from each dilution were used to calculate the CoV, except for the 100-copy number standard, where only five were used due to undetermined values from the assays. The CoV was calculated using the “pastecs” package in RStudio (22, 23). The coefficient of variance was plotted against log10 copy number of the standards (Fig. 2).

The analytical specificity of the real-time PCR assay was assessed using a series of DNA samples from a variety of strains of *Histoplasma* along with two non-target controls described in Table 1. Each DNA sample was diluted to have a final DNA concentration of 20 ng/µL, which was equivalent to 40 ng per qPCR reaction. All strains were run with a standard curve between 10^8^ copies and 100 copies.

**TABLE 1:** *Histoplasma* control DNA and off-target fungal species used

Strain collection number	Species	Origin	Ct value	Result
CBS 176.57^*d*^	*Histoplasma capsulatum* var. *farciminosum*	Unknown	13.80	Positive
CBS 137.72^*d*^	*Histoplasma capsulatum* var. *capsulatum*	Arkansas	16.56	Positive
CBS 537.84^*d*^	*Histoplasma capsulatum* var. *farciminosum*	Egypt	14.24	Positive
CBS 536.84^*d*^	*Histoplasma capsulatum* var. *farciminosum*	Egypt	16.16	Positive
CBS 478.64^*d*^	*Histoplasma capsulatum* var. *farciminosum*	Poland	14.01	Positive
H90 - ATCC 58332^*a*^	*Histoplasma capsulatum* var. *farciminosum*	Egypt	11.70	Positive
G217B^*b*^	*Histoplasma capsulatum*	NAm2	13.81	Positive
G186A^*b*^	*Histoplasma capsulatum*	Panama	12.66	Positive
504^*c*^	*Aspergillus nidulans*	Unknown	36.15	Negative
JGI-competent yeast^*c*^	*Saccharomyces cerevisiae*	Unknown	37.76	Negative

^*a*^ Genomic DNA derived from an equid *Histoplasma* strain supplied by collaborators at Ohio State University.

^*b*^ *Histoplasma capsulatum* reference strain genomic DNA supplied by collaborators at Ohio State university.

^*c*^ *Aspergillus nidulans* and JGI-competent yeast supplied by Mark Caddick at The University of Liverpool.

^*d*^ Westerdijk Fungal Biodiversity Institute.

### Receiver operating characteristics curve

Receiver operating characteristics (ROC) curve analysis was used to fulfill two objectives: (i) to assess the performance of the qPCR assay compared with an alternative method capable of diagnosing clinical cases of EZL (nested ITS PCR) and (ii) to determine a statistically justified Ct cut-off value. Data analysis was conducted using the packages “pROC” and “OptimalCutpoints” in RStudio (23–25). The ROC curve used nested ITS PCR diagnostic data (binomial distribution) from pus samples taken from confirmed positive clinical cases in Senegal and The Gambia as the response variable. The mean Ct values from the same Senegalese and Gambian sample replicates were used as the predictor variable. The ROC curve was plotted using 95% confidence intervals and visualized graphically using “ggplot2” package in RStudio (26). The cut-off point for the Ct value was established with “OptimalCutpoints” package, selecting Youden’s index as the optimizing method in order to obtain the maximum sum of sensitivity and specificity that corresponds to a given Ct cut-off value (27). Once a cut-off value was obtained, a 2 × 2 table was used to calculate the accuracy, misclassification rate, false-positive rate, false-negative rate, diagnostic specificity, diagnostic sensitivity, positive predictive value, and negative predictive value.

### Blood sample cut-off values

Blood samples from all four countries were included in this portion of the analysis. The cut-off values of blood samples could not be analyzed using an ROC curve due to a lack of nested ITS PCR-positive samples among any of the blood samples. A Pearson’s correlation coefficient was performed on paired Ct values of blood and pus samples from The Gambia and Senegal (*n* = 12). No significant correlation was observed, suggesting that inferring the cutoff for blood samples from pus samples would be inappropriate. Thus, an alternative means of establishing a cut-off value for the blood samples was used. The cutoff was determined using the mean Ct value of the 1,000 copy/reaction standards (10 replicates) as this was the lowest copy number standard that was detectable in all 10 repeats.

### Examining the limit of detection in equine blood samples

To assess the performance of the *Histoplasma* DNA extraction in blood from Whatman FTA cards (GE Healthcare), horse blood in 1.5 mg/mL EDTA (TCS Biosciences Ltd, Catalog number: HB073) was prepared in 1,000 µL volumes in sterile 1.5 mL tubes. Genomic DNA from HCF isolate H90 was diluted in nuclease-free water to 10^6^ copies/µL. A 1:10 serial dilution from 10^6^ to 10^2^ copies/µL was made. From the five dilutions, 10 µL of each dilution was pipetted into the 1,000 µL of horse blood in duplicate. Samples were vortexed for 10 s followed by 200 µL of sample being inoculated directly onto the Whatman FTA cards. The Whatman FTA cards were left to air dry overnight in a UV sanitized class II cabinet. Once dry, the cards were microwaved at ~900 W for 30 s, left to stand for 1 min, then microwaved for a further 30 s as described in reference (8). The Whatman cards were stored at 4°C, until DNA was extracted from the cards using the DNA extraction method as described in reference (8).

The DNA extraction method was followed exactly, except for steps referring to the cutting out of card pieces; instead, 100 µL of inoculated blood was added directly into a sterile 2-mL screw cap tube containing 0.5 g 425–600 µm acid-washed glass beads (Sigma Life Sciences, Catalog number: G8772-500G).

Paired *t*-tests were used to assess differences in Ct values between the FTA card groups and direct extraction groups. A performance comparison between the methods was conducted by comparing the percentage of DNA recovered from FTA cards to that recovered from whole blood samples.

### Clinical data analysis

Clinical examination metadata were available for horses and donkeys sampled in The Gambia, and positive and negative samples were identified using the qPCR cut-off points described. Any samples with missing data points for the clinical variable being analyzed were omitted before testing. Univariable analyses were conducted in R Studio (22) to explore the Ct values obtained and for associations with sample type, equine species, disease severity, and differential cell count. Wilcoxon rank-sum tests were used to compare Ct values obtained from blood and pus samples collected from The Gambia (Fig. 6) and to explore differences in the Ct values of blood and pus samples obtained from different equine species.

Clinical cases were categorized into “Mild,” “Moderate,” or “Severe” as described in reference (8). As there was only one moderate case among this data set, it was merged with the severe category. The mean Ct values obtained from pus and blood samples were compared among cases categorized as severe or mild using Wilcoxon rank-sum tests.

Associations between presence/absence of *Histoplasma* by qPCR test and blood count parameters were explored using a Pearson’s correlation coefficient or Spearman’s rank correlation (as appropriate to the data distribution). Differential white blood cell counts of major cell lines were investigated, including neutrophils, basophils, eosinophils, monocytes, and lymphocytes (Table S3).

## RESULTS

The optimized qPCR assay detected several strains of *Histoplasma* (8/8, 100%), while off-target fungal species (0/2, 0%) were undetected (Table 1). All *Histoplasma* strains in the reactions amplified with a mean Ct value of 14.12, whereas *Aspergillus nidulans* and *Saccharomyces cerevisiae* both had one undeterminable result and one amplification with Ct values of 36.15, and 37.76, respectively. These were interpreted as negative due to the Ct values exceeding cutoff calculated among the blood and pus samples.

A BLAST search of the three (3/3) *Histoplasma capsulatum* var. *farciminosum* strains (AB055249.1, AF322387.1, and AB071838.1) in NCBI’s non-redundant (nr) database showed 100% sequence identity to the probe and the internal transcribed spacer 1 region.

### Analytical sensitivity

The analytical sensitivity of the assay was determined to be 1,000 copies/reaction. This was chosen as it was the lowest standard to have a coefficient of variance less than 5% for which all 10 standards were quantifiable (Fig. 2). The graph depicts a trend of higher CoV values the higher the concentration of the standards.

### Receiver operating characteristics

**Fig 3:**
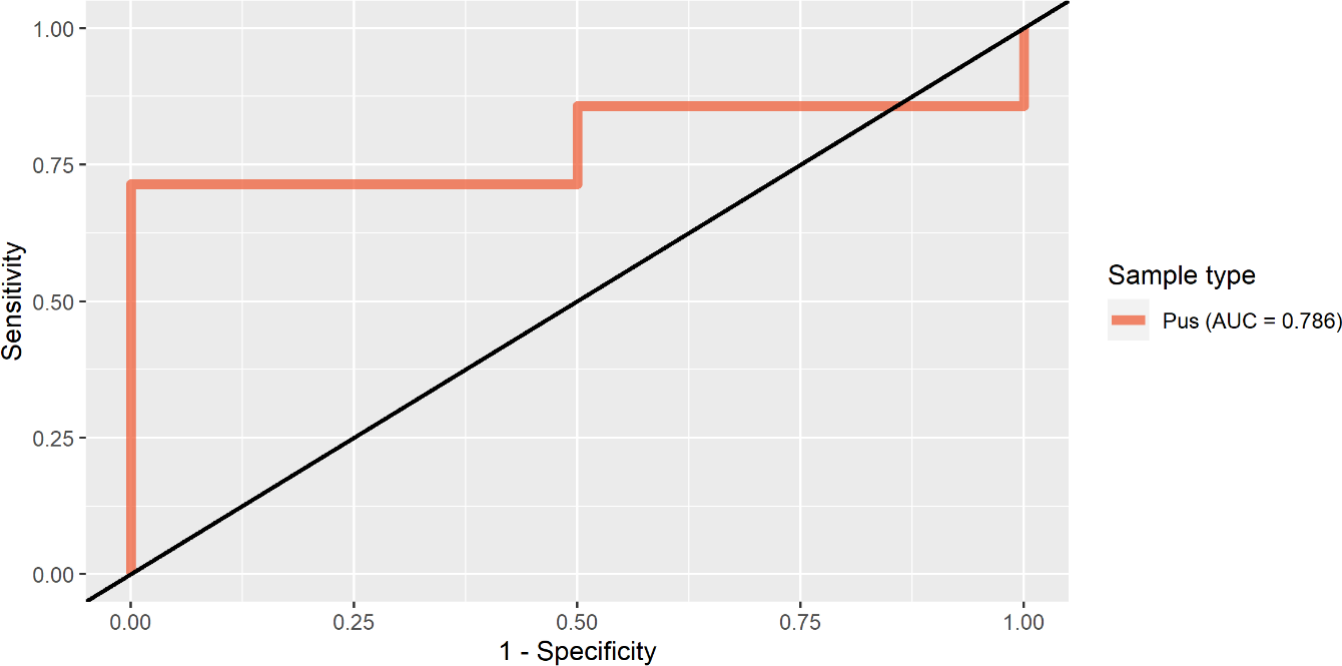
An ROC curve demonstrating the performance of the qPCR method compared with a nested ITS method. The red line indicates assay performance with an area under the curve (AUC) of 0.786.

Results from an ROC curve conducted on pus samples from The Gambia (*n* = 13) and Senegal (*n* = 3) display the performance of the qPCR method in comparison with nested ITS PCR (Fig. 3) (ITS nested PCR results available in supplementary tables). The main derivative from the ROC curve analysis was a cut-off Ct value of 27.75 which was defined using Youden’s index. A 2 × 2 contingency table was generated from the Youden’s index cut-off value revealing a false-negative and false-positive rate of 25% and 0%, respectively. The accuracy of the qPCR method was 75%, and the misclassification rate was 25%. According to the cut-off value proposed, the specificity was 100% and the sensitivity 71.4%. The positive predictive value and negative predictive value were 100% and 33%, respectively. Ten pus samples tested positive (DNA copy number; *n* = 10, min = 56,513, max = 324,360, range = 267,848, median = 107,342) according to the Youden’s index cut-off value and six tested negative (DNA copy number; *n* = 6, min = 6,678, max = 44,089, range = 37,411, median = 17,671). The ROC curve demonstrates high performance of the qPCR when compared to nested ITS PCR method (Fig. 3).

### Blood sample cut-off values

**Fig 4:**
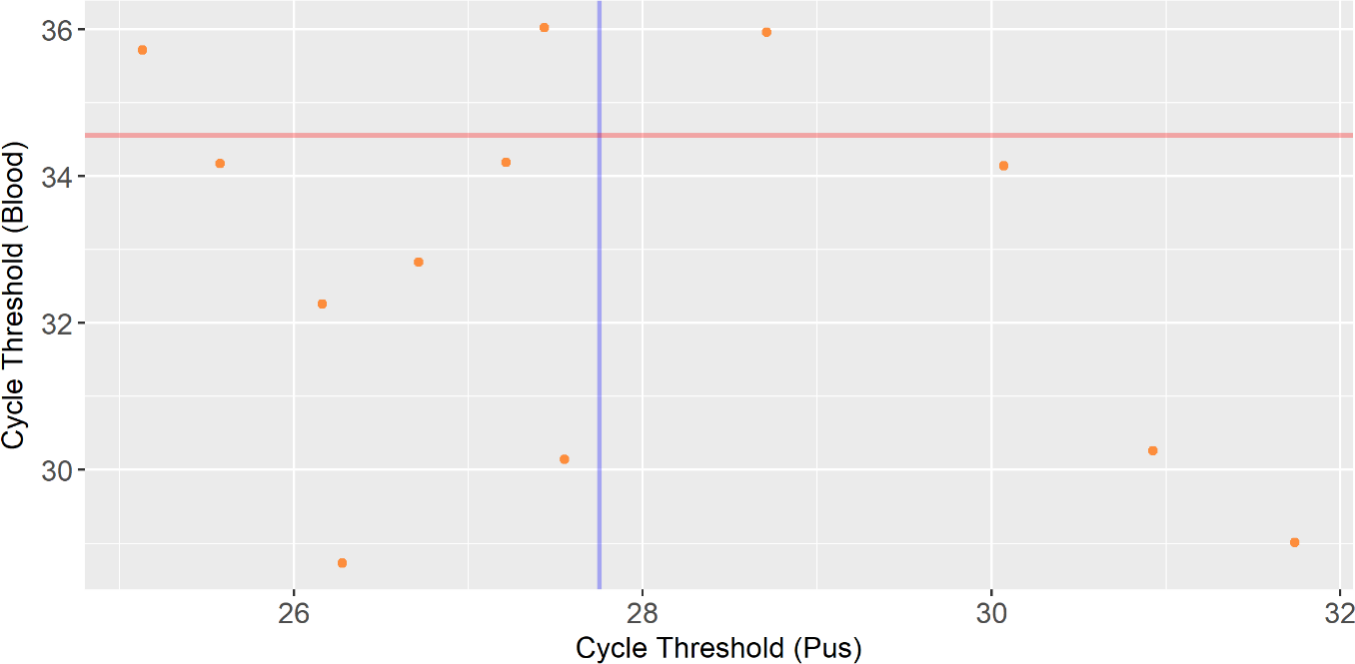
An analysis of a correlation between the Ct values of 12 paired pus and blood samples from 9 horses and 3 donkeys in The Gambia. The graph shows the relationship between the Ct values of blood and pus. No significant relationship was found between positive blood and positive pus samples. The blue vertical line represents the Youden’s index cut-off value in pus (27.75) determined by the ROC curve in Fig. 3. The red horizonal line represents the mean Ct value of standards used to determine the limit of detection (34.55).

A Pearson’s correlation analysis was conducted to determine whether there was a significant relationship between the Ct values of paired blood and pus samples. No significant relationship was found between paired blood and pus samples (*P*-value = 0.26) (Fig. 4). An alternative approach was taken to try to determine the optimal cut-off value. The mean Ct value of the limit of detection (1,000 copies/reaction, Ct = 34.55) was used as the diagnostic cutoff for blood samples. Using the proposed blood cut-off value, three sample sets were analyzed for their proportions of positive results. Samples from Ethiopia that were presumed to be negative for EZL revealed 13 positive results and 2 negative results (13/15, 87%; positive DNA copy numbers: *n* = 13, min = 1,355, max = 78,051, range = 76,696, median = 3,601; Ct Values: *n* = 13, min = 27.93, max = 34.20, range = 6.27, median = 32.68). Blood samples from Senegal and The Gambia when combined showed 15 positive results and 5 negative results (15/20, 75%; positive DNA copy number: *n* = 15, min = 661, max = 72,893, range = 72,232, median = 8,138; Ct values: *n* = 15, min = 28.73, max = 34.19, range = 5.46, median = 32.83). With samples from Mali, 13 samples tested positive and zero tested negative (13/13, 100%; positive DNA copy number: *n* = 13, min = 727, max = 18,947, range = 18,221, median = 1,506; Ct Value: *n* = 13, min = 30.46, max = 34.47, range = 5.46, median = 33.58).

### DNA extraction yields between FTA cards and whole blood

**Fig 5:**
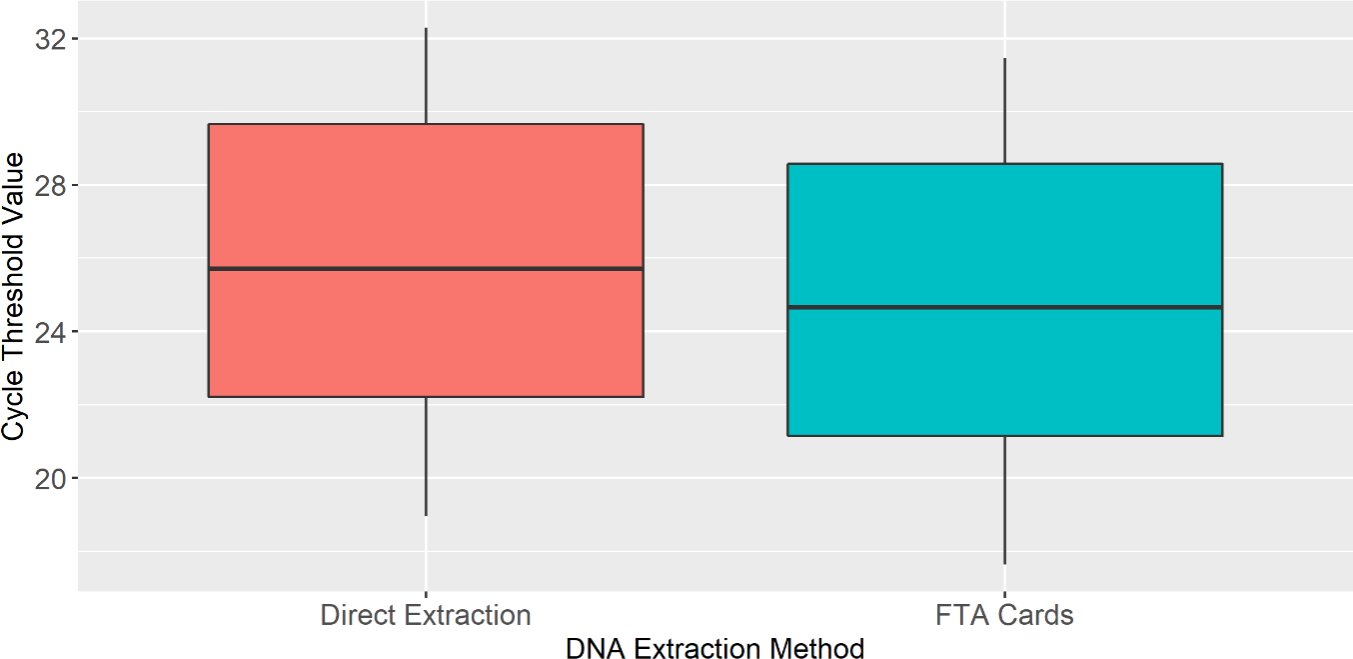
Box and whisker plot comparing DNA yield (Ct value) between direct extraction (red) and FTA cards (blue) obtained from blood spiked with serial dilutions of *Histoplasma* genomic DNA. These data show that FTA cards spiked with blood (*n* = 20, mean = 24.65, SD = 4.90) consistently yield lower Ct values than DNA extracts prepared directly from whole blood spiked with genomic *Histoplasma* (*n* = 20, mean = 25.81, SD = 4.73 paired *t*-test *P*-value <0.001).

Whatman FTA cards were assessed by conducting a paired *t*-test to compare DNA yield direct from blood with DNA extraction of blood on FTA cards. The paired *t*-test showed a significant mean difference (*t* = 15.614, df = 19, *P*-value <0.001) when comparing the Ct values of Whatman FTA cards with blood (*n* = 20, mean = 24.65, SD = 4.90) and DNA extracts directly from blood (*n* = 20, mean = 25.81, SD = 4.72) (Fig. 5), demonstrating a higher yield of DNA from the Whatman FTA cards than directly from blood. Percentage recovery between the two methods ranged from 23.31% to 41.78% with a mean difference of recovery of 34.68% across five different dilutions, demonstrating that direct extraction recovers only approximately 34.68% of the DNA that Whatman cards recover.

### Blood and pus sample comparison

**Fig 6:**
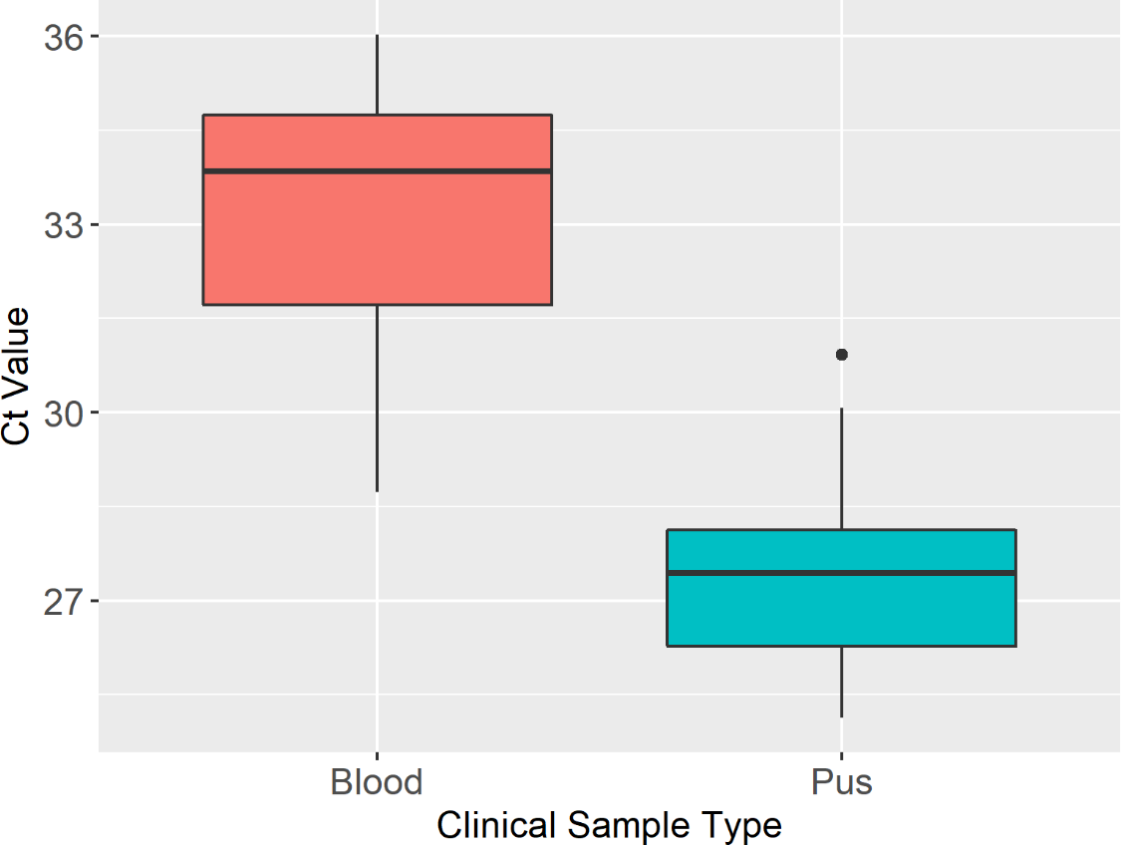
Comparing Ct values obtained from clinical blood and pus samples from The Gambia. Distribution of Ct values of blood and pus samples from 16 horses and 3 donkeys. Ct values in blood (*n* = 19, min = 28.73, max = 36.02, median = 33.85, range = 7.29; donkeys = 3 and horses = 16) and pus (*n* = 13, min = 25.13, max = 30.92, range = 5.79, median = 27.44; donkeys = 3 and horses = 10). The dot outside of the box and whisker plot represents one outlying data point.

Overall Ct values obtained from blood and pus samples from 19 equids from The Gambia revealed significantly higher Ct values in blood compared to pus samples (Wilcoxon rank-sum test *P* < 0.001) (Fig. 6).

### Comparison of Ct values and clinical parameters

**Fig 7:**
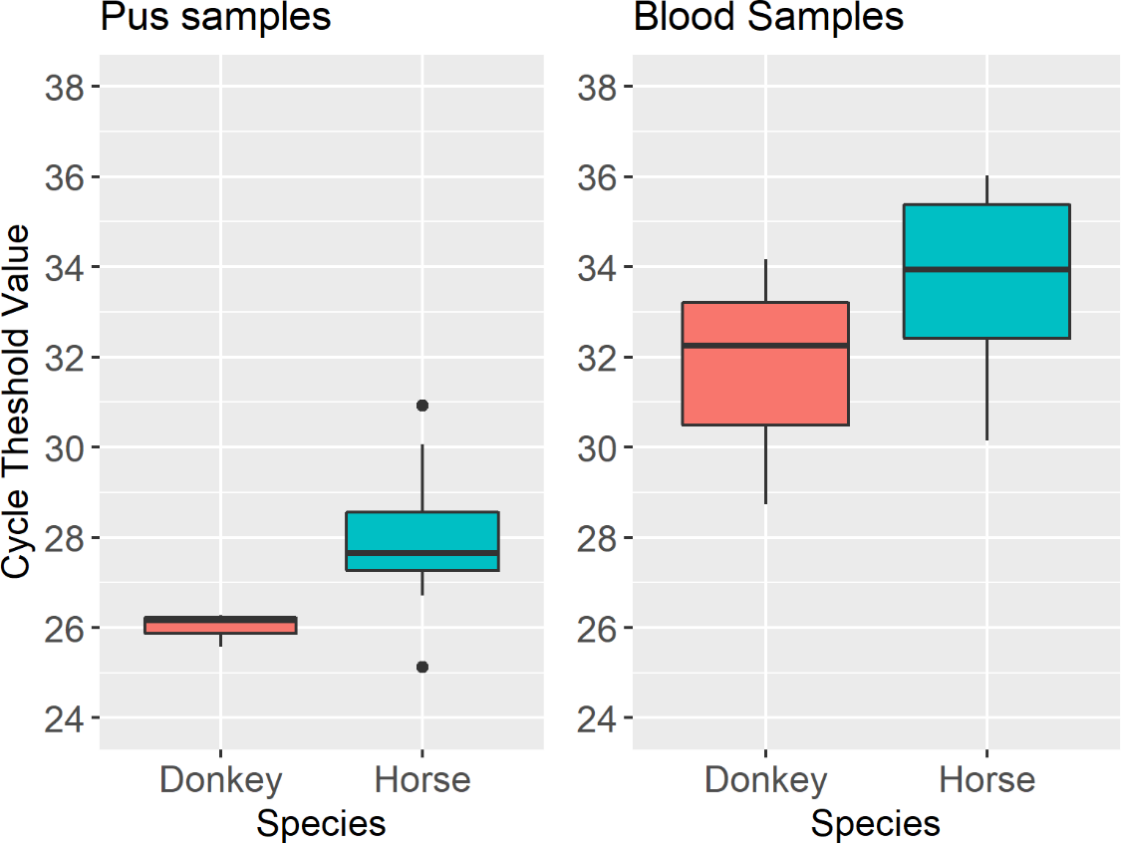
Box and whisker plots of the distribution of Ct values obtained from blood and pus samples from horses and donkeys with EZL. Left hand panel: Distribution of Ct values obtained from pus samples from 3 donkeys and 10 horses from The Gambia. Pus samples from donkeys yielded lower Ct values than pus samples from horses (Ct values among donkeys: min = 25.58, max = 26.28, median = 26.16, range = 0.70 and horses: min = 25.13, max = 30.92, median = 27.65, range = 5.79). The dots outside of the box and whisker plot represent outlying data points. Right hand panel: Distribution of Ct values obtained from blood samples from 3 donkeys and 16 horses from The Gambia. Although the median Ct values for blood samples were generally lower in donkeys compared to horses, this was not statistically significant (Wilcoxon rank-sum test *P*-value = 0.303). The comparison of the Ct values between donkeys (Ct values from donkeys: mean = 31.72, SD = 2.76 and horses: min = 30.14, max = 36.02, range = 5.88, median = 33.94).

A Wilcoxon rank-sum test comparing the differences in the Ct values of pus samples between donkeys and horses revealed a significant difference (*W* = 3, *P*-value <0.05) (Fig. 7). Donkeys appear to have significantly lower mean Ct values in pus samples (*n* = 3, min = 25.58, max = 26.28, median = 26.16, range = 0.70) than horses (*n* = 10, min = 25.13, max = 30.92, median = 27.65, range = 5.79). There was no significant difference in Ct values obtained from blood samples from different species (Fig. 7).

### qPCR results and clinical presentation

**Fig 8:**
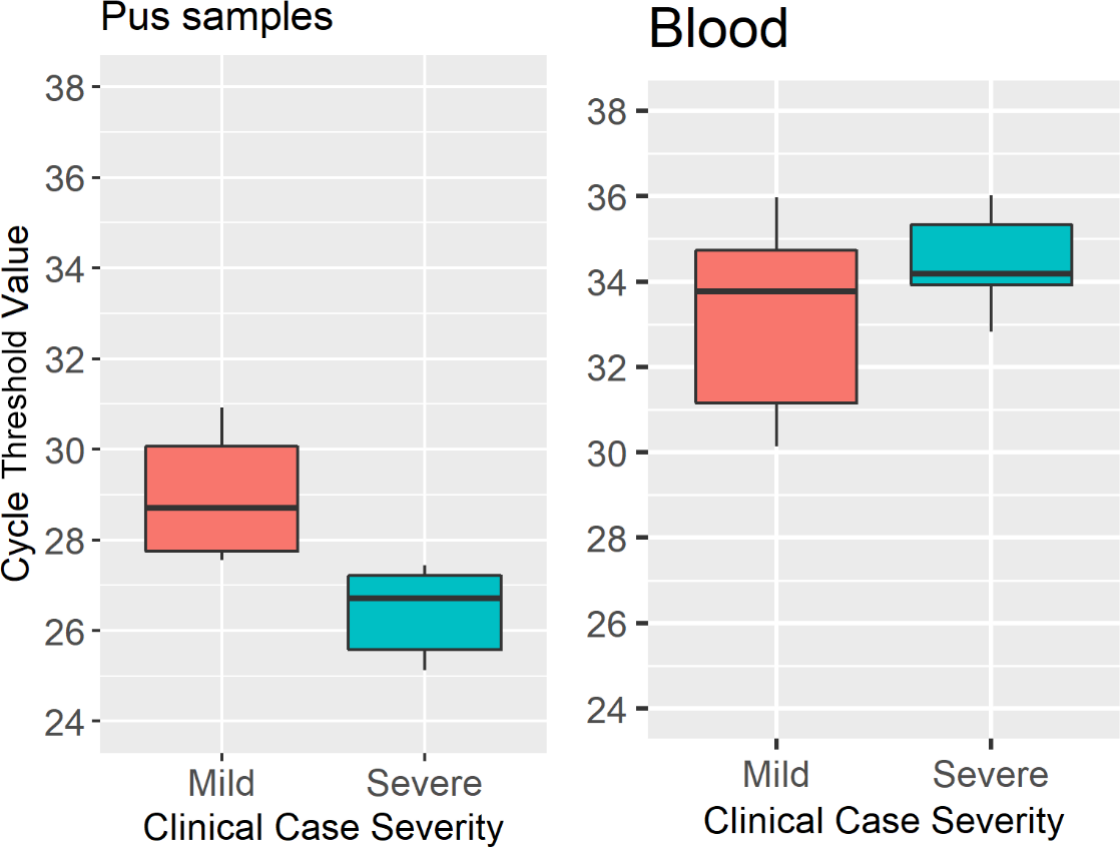
Comparison of distribution of Ct values obtained from pus and blood samples among mild (red) and severe (blue) cases of EZL. Left hand panel: Comparison of Ct values obtained from pus samples from nine horses and one donkey with EZL. Mild cases of EZL were significantly associated with higher Ct values than severe cases (Wilcoxon rank-sum *P* = 0.007). (Distribution of Ct values in mild cases: median = 28.71, min = 27.55, max = 30.92; distribution of CT values in severe cases of EZL: median = 26.72, min = 25.13, max = 27.44.) Right hand panel: Comparison of Ct values obtained from blood samples from 16 horses and 1 donkey with EZL. Those with mild disease (*n* = 11) tended to yield lower Ct values than those with severe disease (*n* = 5); however, this was not statistically significant Wilcoxon rank-sum *P* = 0.18. Ct values obtained from blood samples from mild (median 33.76, min = 30.15, max = 35.97) and severe cases (median 34.19, min = 32.83, max = 36.02).

Ct values obtained from pus samples from 10 equids from The Gambia presenting with mild EZL were significantly different to those obtained from severe cases of EZL (Wilcoxon rank-sum test *P* = 0.007) (Fig. 8). This was not apparent in blood samples from the same animals where there was no significant difference observed between Ct values from mild and severe cases Wilkcoxon rank-sum *P* = 0.18 (Fig. 8). Out of a total 10 positive pus samples from The Gambia and Senegal, as interpreted by the cut-off point based on Youden’s index, 5 were classified as severe (DNA copy number: median = 80,741, min = 70,303, max = 324,361, range = 254,057), 2 were mild (DNA copy number: median = 60,808, min = 56,512, max = 65,103, range = 8,591), and 3 did not have data for severity (DNA copy number: median 150,983, min = 44,088, max = 165,529). Of the qPCR-negative cases, all were either mild (DNA copy number: *n* = 3, median = 12,033, min = 6,834, max = 29,822, range = 22,988) or had no data recorded on case severity (DNA copy number: *n* = 3, median = 23,310, min = 6,678, max = 44,089, range = 37,411).

### Differential cell count analysis

A negative correlation was observed between basophil counts and the Ct values from paired pus samples, where an increased presence of *Histoplasma* in pus demonstrated by a lower Ct value (median = 27.44, min = 25.13, max = 30.92) was associated with a higher basophil count (median = 3, min = 0, max = 9) (Spearman’s rank correlation analysis *P* = 0.005). No other significant relationships between cell counts of other cell lineages and Ct value were observed in either blood or pus samples. However, it is of note that all cases from The Gambia with a full differential cell count (*n* = 15) demonstrated a monocytosis (median 42%) (Table 2; Table S3).

**TABLE 2:** Differential cell count^*a*^ ranges among 15 horses sampled in The Gambia

Cell type	Cell count ranges in study horses	Median cell count	Reference range in horses
Band neutrophils	0%–12%	2	0%–1%
Segmented neutrophils	8%–60%	26	30%–75%
Basophils	0%–9%	2	0%–3%
Eosinophils	0%–10%	3	1%–10%
Monocytes	10%–64%	42	1%–8%
Lymphocytes	1%–50%	20	25%–60%

^*a*^ Based on manual differential cell counting of proportions within 100 cells.

## DISCUSSION

This is the first study to demonstrate the ability to detect the DNA of *Histoplasma capsulatum* var. *farciminosum* directly from equine blood and pus samples, offering a sensitive method to diagnose and quantify infection burden. This study has evaluated the utility of primers and probes targeting the ITS region (13) for detection of both HCC and HCF. These findings demonstrate that this qPCR protocol is sufficient to provide a yes/no diagnostic for the presence of *Histoplasma* at the genus level and encompasses both HCC and HCF; however, it does not differentiate between *Histoplasma* sub-species. Although a few studies have recognized the diversity among the genus *Histoplasma* (28–30) including notably, the identification of a number of sub-species of HCF (8, 30), a lack of genomic data for varieties of HCF originating from recent specimens from the African continent impedes the design of more specific primers to enable species differentiation. Further elucidation of the genome of HCF is warranted to enable enhanced targeting of gene loci for diagnostics and improve understanding of the pathogenesis of HCF. In particular, genome sequencing and analysis could reveal gene loci coding for specific transcription factors involved in assembly of proteins that are required for host immune evasion or adaptation of the pathogen during the blood-borne phase of the disease that we have detected in this data set.

There are variations in the way detection limits for a qPCR reaction are reported in the literature. Some studies report the minimum amount of analyte that is detectable in a qPCR reaction (13). Other studies report the limit of detection as the minimum amount of recoverable DNA that can be obtained from a matrix of microbial culture/standard and negative clinical specimen (14, 15). However, this does not account for inter-assay variability and the consistency of detection. Therefore, an approach factoring the detectability of plasmid standards with a given cut-off CoV value was used to report a limit of detection. A limit of detection was calculated using the CoV of Ct values from a series of standards ranging from 10^8^ to 100 copies in five assays (10 replicates). The lowest point where all 10 standards elicited a result from the qPCR assay with a CoV of less than 5% was 1,000 copies/reaction. However, there was a positive association between the CoV of standards and their respective copy number. An ideal standard would have a CoV of less than 5% at all dilutions. Our standard has a CoV greater than 5% but less than 10% at 10^5^, 10^6^, and 10^7^, so we propose a linear dynamic range of 1,000 copies/reaction and 10^7^ copies/reaction.

While exploring multiple facets of the performance of the assay, the method successfully detected a variety of *Histoplasma* spp. in pus samples with encouraging conformity to an established nested ITS PCR method (Table S1). The qPCR successfully detected 100% (8/8) of the control strain *Histoplasma* spp. and 0% (0/2) of other fungal species.

An ROC curve was used to determine an optimal Ct cut-off value (27.75), providing a model capable of diagnosing clinical cases of EZL with 100% specificity and 71.4% sensitivity. Although the proposed cut-off value provides high diagnostic specificity and sensitivity, one limitation is the lack of a clinical control group. All pus samples used to validate the methods performance and cutoff were taken from clinical cases presumed to have EZL. This is supported by the small number of pus samples used to validate the cutoff. Only two tested negative using the nested ITS PCR method (2/16, 12.5%). With a lack of negative cases to include in the validation, as horses with no clinical signs of EZL had no pus samples present, it can be challenging to identify the true threshold at which a pus sample would be negative. Other qPCR studies targeting *Histoplasma* spp. show varying diagnostic sensitivities and specificities ranging from 73% to 96% and 95.4% to 100% (13–15). Although these sensitivities and specificities were reported in different clinical specimens to those used in this study, this assay, when used to diagnose clinical cases using equine pus, displays similar performance to other qPCR assays (13–15).

It is not anticipated that any storage depletion effects would have occurred for any of the samples used in this study, as samples on FTA cards are inherently dried, fixed, and stabilized. DNA extraction was carried out directly from intact FTA cards immediately prior to samples being tested using qPCR. The samples from The Gambia, Mali, and Senegal were collected on FTA cards during 2016, the blood sample FTA card spots were collected from Ethiopia during 2013, and all were processed for this study during 2017. Samples of fungal culture have been shown to be viable up to 18 months post inoculation onto FTA cards (31), and blood spots on FTA cards remain stable for extended periods even greater than 15 years (32).

Diagnosing *Histoplasma* using blood samples has been demonstrated to be possible in this study; however, it is acknowledged that further optimization is required to provide a more representative cut-off point that may be applied to wider populations. This study lacked negative controls for blood samples which impeded the ability to create an ROC curve model for accurate detection threshold of *Histoplasma* spp. in the blood. However, we have demonstrated that it is possible to detect *Histoplasma* among equine blood samples from clinical and asymptomatic cases and that it is possible to detect and recover HCF DNA both from direct extraction from blood and blood spiked with DNA onto Whatman FTA cards. The mean Ct value of the standards used to establish the detection limits of the assay (34.55) was used as our cutoff. Using this value as a diagnostic cutoff gave positive rates of infection within groups of blood samples from Senegal and The Gambia (Senegal and The Gambia are grouped), Ethiopia, and Mali of 75% (15/20), 87% (13/15), and 100% (13/13), respectively. However, the lack of a correlation between the Ct values of paired pus and blood samples indicated that basing the cutoff for blood samples on the data generated from pus samples would not provide reliable results. Reasons for the observed differences in Ct values between blood and pus cut-off values may be due to (i) fungemia being thought to be transient in the blood (33), (ii) levels of *Histoplasma* in the blood being different at various stages of infection (as some of our data supports this hypothesis), and (iii) *Histoplasma* load being significantly higher in pus than in blood.

While samples from Mali, Senegal, and The Gambia were all presumed positive cases based on clinical presentation, microscopic, and nested PCR results, samples from Ethiopia demonstrated varying results. Blood samples from Ethiopia originated from horses with no clinical signs of EZL here were reported positives among these samples that could represent either early or asymptomatic cases, corroborating findings from our previous study (8). This adds weight to the possibility of utilizing this technique to explore the clinical phenomenon of asymptomatic carriage, which could unlock important epidemiological information about the timing and progression of clinical signs and transmission of disease.

This study demonstrates, for the first time, a differentiation in the infection burden across a series of equine EZL cases. Although conclusions should be made with caution due to this being based upon a small sample size, there are compelling differences apparent between the amount of *Histoplasma* cells detectable in pus compared to blood samples, among different equine species, and clinical case presentations. Overall, lower Ct values were consistently demonstrated across pus samples compared with those obtained from blood samples. This seems intuitive, given the primary location of the clinical lesions being concentrated in the lymphatic system and expressed within pyogranulomatous nodules. This does, however, indicate that cases with clinical signs, including skin lesions, did also demonstrate the presence of *Histoplasma* within the blood stream, demonstrating the role of the circulatory system in distributing the pathogen to other body sites and systems in advanced cases.

A previous study by this group (8) reported the ability to detect *Histoplasma* in equine blood samples using nested PCR, and hypotheses were formulated about the significance of this during the clinical development of a case. Questions were raised whether this would be useful in detecting asymptomatic carriers and/or of practical benefit to facilitate early detection in blood. Although these data originate from a cross-sectional study, meaning no inference on timing, or sequence of appearance of *Histoplasma* in the blood stream can be made, the findings here highlight the ability to detect and quantify *Histoplasma* in the blood and suggest a difference between equine species and mild and severe cases.

When breaking down the analysis into mild and severe cases, the trend in detection of *Histoplasma* in pus and blood samples was varied. In severe cases, presenting with extensive lesions in more than one body area (as per the grading system used), there was a significantly higher quantity of *Histoplasma* cells in pus samples compared with blood. Whereas, in mild cases, this was inversed, and, although not statistically significant, the trend demonstrated a higher quantity of *Histoplasma* cells in blood compared to pus in mild cases. This may provide some insight into the sequence of detection of pathogen in blood and pus samples among mild and severe cases and could indicate that *Histoplasma* circulates within blood for some time prior to the appearance of clinical signs. This is an area that warrants further investigation, as it could indicate an ability to detect cases early in the course of infection, which is critical in reducing treatment costs, duration, and improving patient outcome. The data set from Ethiopia comprised animals with no external clinical signs of EZL; however, *Histoplasma* was detectable among some of these blood samples, a finding that was also reported in our previous study (8). This requires further investigation in a wider population to investigate the significance of these findings and to measure the scale of this phenomenon among representative clinical cases and control animals. Additional focused investigation around early stages of infection could assist with understanding the role of asymptomatic carriers within an endemic population, another key area to target when considering disease control strategies.

These data present the first confirmation of EZL in donkeys in The Gambia; EZL has previously been reported sporadically in donkey populations in Ethiopia (34, 35). Findings in this study suggest that donkeys with clinical signs have higher pathogen load compared with horses with similar signs. Although firm conclusions cannot be made with the scale of this data set, it perhaps supports a hypothesis that donkeys may be more resistant to EZL than horses, as clinical disease is not frequently reported among donkeys (*pers. comms*), only sporadically in endemic regions. One hypothesis could be that if indeed donkeys are presenting clinical signs, then this may be because they have a particularly high infection burden or a particular strain of *Histoplasma* that is more pathogenic for donkeys. The role of donkeys as possible asymptomatic carriers among mixed equine populations is an area that requires investigation.

It is notable that differential neutrophil counts across all clinical cases were deemed to be within low or normal range, while basophils were either high or normal. This may be indicative of overwhelming infection or suggest an immune response is mounted similar to that in response to a parasitic infection. This warrants further investigation to characterize and understand the equine immune response throughout the cycle of infection and disease. Generally, all cases demonstrated monocytosis with average counts of 40%, demonstrating a greater than fourfold increase compared with the normal range. This is indicative of chronic infection that stimulates cell-mediated immunity and agrees with findings from our previous study reported from cases in Ethiopia (8).

These quantitative findings provide promising insights into the utility of this qPCR method to examine the pathogenicity and elucidate the epidemiology of this disease. Currently, facilities to carry out qPCR within the countries from which these samples originated are limited. Although the overall cost of PCR and qPCR has progressively reduced over recent times, there is yet to be a significant increase in the use of these methods in these regions. Sustainable funding, technical capacity, and infrastructure are required to support the development of molecular diagnostics within these areas. Such techniques would have the capacity to rapidly advance understanding of regional endemic diseases, leading to greater ability to support surveillance and control programs to promote animal health.

In conclusion, this study demonstrates the ability to detect *Histoplasma* directly from equine clinical samples using qPCR and offers a powerful tool to investigate the epidemiology of this disease. The ability to detect and quantify *Histoplasma* in blood delivers quantitative information on pathogen load with scope to further investigate the role of fungemia in infection and asymptomatic carriage within endemic populations.

## SUPPLEMENTAL MATERIAL

Supplemental material spectrum.03100-23-s0001.docx Tables S1 to S3.

ASM does not own the copyrights to Supplemental Material that may be linked to, or accessed through, an article. The authors have granted ASM a non-exclusive, world-wide license to publish the Supplemental Material files. Please contact the corresponding author directly for reuse.
