# Supplementary material for: Validation of a real-time polymerase chain reaction for the detection and quantification of the nucleic acid of Histoplasma from equine clinical samples
Source: Microbiol Spectr. 2024 Feb 27;12(4):e03100-23. doi: 10.1128/spectrum.03100-23 (PMC10986522; doi:10.1128/spectrum.03100-23)
Supplement: Supplemental material — Tables S1 to S3. [file spectrum.03100-23-s0001.docx]

**Supplementary table 1.** Table of average cycle threshold values, mean copy numbers and ITS PCR result from clinical samples used in this study

| **Sample ID** | **CT Mean^1^** | **CT Standard Deviation** | **Copy number Mean^2^** | **Copy Number Standard Deviation** | **Country** | **Clinical Sample** | **ITS PCR Result^3^** | **qPCR Result** |
| --- | --- | --- | --- | --- | --- | --- | --- | --- |
| ANK01P | 26.72 | 0.11 | 80270 | 5499 | Gambia | Pus | 1 | 1^a^ |
| DB01P | 30.07 | 0.00 | 12033 | 3 | Gambia | Pus | 0 | 0 ^a^ |
| DB02P | 26.16 | 0.37 | 165529 | 40638 | Gambia | Pus | 1 | 1 ^a^ |
| DB03P | 27.75 | 0.07 | 56513 | 2477 | Gambia | Pus | 1 | 1 ^a^ |
| KT01P | 27.55 | 0.28 | 65103 | 12299 | Gambia | Pus | 1 | 1 ^a^ |
| KTB01P | 25.13 | 0.02 | 324360 | 5252 | Gambia | Pus | 1 | 1 ^a^ |
| KTB02P | 27.22 | 0.05 | 80741 | 2910 | Gambia | Pus | 1 | 1 ^a^ |
| KTB04P | 28.71 | 0.00 | 29822 | 70 | Gambia | Pus | 1 | 0 ^a^ |
| KTKL1P | 27.44 | 0.27 | 70303 | 12614 | Gambia | Pus | 1 | 1 ^a^ |
| KTKL2P | 26.28 | 0.07 | 150983 | 7480 | Gambia | Pus | 1 | 1 ^a^ |
| KTKL4P | 30.92 | 0.21 | 6834 | 946 | Gambia | Pus | 1 | 0 ^a^ |
| KTKL5P | 25.58 | 0.00 | 241323 | 31 | Gambia | Pus | 1 | 1 ^a^ |
| KTKL6P | 28.12 | 0.07 | 44089 | 2013 | Gambia | Pus | 0 | 0 ^a^ |
| ANK01B | 32.83 | 0.14 | 1910 | 175 | Gambia | Blood | 0 | 1^b^ |
| DB01B | 34.15 | 0.50 | 8138 | 2922 | Gambia | Blood | 0 | 1^b^ |
| DB02B | 32.26 | 0.43 | 32109 | 9986 | Gambia | Blood | 0 | 1^b^ |
| KT01B | 30.15 | 0.11 | 11438 | 865 | Gambia | Blood | 0 | 1^b^ |
| KTB01B | 35.72 | 0.16 | 247 | 26 | Gambia | Blood | 0 | 0^b^ |
| KTB02B | 34.19 | 0.15 | 661 | 66 | Gambia | Blood | 0 | 1^b^ |
| KTB03B | 33.85 | 0.45 | 10026 | 3238 | Gambia | Blood | 0 | 1^b^ |
| KTB04B | 35.97 | 0.24 | 212 | 33 | Gambia | Blood | 0 | 0^b^ |
| KTB05B | 33.76 | 0.45 | 1046 | 313 | Gambia | Blood | 0 | 1^b^ |
| KTB06B | 35.58 | 0.09 | 304 | 19 | Gambia | Blood | 0 | 0^b^ |
| KTKL1B | 36.02 | 0.42 | 207 | 55 | Gambia | Blood | 0 | 0^b^ |
| KTKL2B | 28.73 | 0.08 | 21953 | 1197 | Gambia | Blood | 0 | 1^b^ |
| KTKL3B | 34.02 | 0.17 | 864 | 100 | Gambia | Blood | 0 | 1^b^ |
| KTKL4B | 30.26 | 0.00 | 8223 | 10 | Gambia | Blood | 0 | 1^b^ |
| KTKL5B | 34.17 | 0.24 | 671 | 105 | Gambia | Blood | 0 | 1^b^ |
| MJK01B | 31.13 | 0.42 | 72893 | 22121 | Gambia | Blood | 0 | 1^b^ |
| MJK02B | 35.30 | 0.13 | 366 | 31 | Gambia | Blood | 0 | 0^b^ |
| SNJ01B | 31.17 | 0.14 | 4601 | 403 | Gambia | Blood | 0 | 1^b^ |
| SNJ02B | 33.40 | 0.30 | 1108 | 212 | Gambia | Blood | 0 | 1^b^ |
| SEN1P | 27.06 | 0.21 | 133944 | 18169 | Senegal | Pus | 1 | 1 ^a^ |
| SEN2P | 29.77 | 0.04 | 23310 | 541 | Senegal | Pus | 1 | 0 ^a^ |
| SEN3P | 31.74 | 0.38 | 6678 | 1635 | Senegal | Pus | 1 | 0 ^a^ |
| SEN3B | 29.01 | 0.00 | 18383 | 16 | Senegal | Blood | 0 | 1^b^ |
| m1 | 34.22 | 0.09 | 888 | 63 | Mali | Blood | 0 | 1^b^ |
| m2 | 34.47 | 0.17 | 727 | 99 | Mali | Blood | 0 | 1^b^ |
| m3 | 32.09 | 0.32 | 5120 | 1328 | Mali | Blood | 0 | 1^b^ |
| m4 | 33.18 | 0.26 | 2095 | 439 | Mali | Blood | 0 | 1^b^ |
| m5 | 33.58 | 0.16 | 1506 | 195 | Mali | Blood | 0 | 1^b^ |
| m6 | 33.35 | 0.01 | 1808 | 9 | Mali | Blood | 0 | 1^b^ |
| m7 | 30.46 | 0.07 | 18947 | 1127 | Mali | Blood | 0 | 1^b^ |
| m8 | 34.36 | 0.36 | 809 | 231 | Mali | Blood | 0 | 1^b^ |
| m9 | 34.44 | 0.22 | 745 | 132 | Mali | Blood | 0 | 1^b^ |
| m10 | 31.10 | 0.06 | 11254 | 532 | Mali | Blood | 0 | 1^b^ |
| m11 | 34.00 | 0.11 | 1066 | 94 | Mali | Blood | 0 | 1^b^ |
| m12 | 34.16 | 0.39 | 953 | 297 | Mali | Blood | 0 | 1^b^ |
| m13 | 33.25 | 0.26 | 1975 | 408 | Mali | Blood | 0 | 1^b^ |
| DE1 | 32.88 | 0.15 | 3169 | 312 | Ethiopia | Blood | N/A | 1^b^ |
| DE3 | 35.45 | 0.26 | 610 | 101 | Ethiopia | Blood | N/A | 0^b^ |
| DE6 | 32.68 | 0.17 | 3601 | 395 | Ethiopia | Blood | N/A | 1^b^ |
| SHA6 | 34.69 | 0.36 | 1000 | 227 | Ethiopia | Blood | N/A | 0^b^ |
| SHA12 | 33.52 | 0.20 | 2100 | 264 | Ethiopia | Blood | N/A | 1^b^ |
| WEL2 | 34.20 | 0.07 | 1355 | 61 | Ethiopia | Blood | N/A | 1^b^ |
| WEL3 | 33.80 | 0.46 | 1784 | 518 | Ethiopia | Blood | N/A | 1^b^ |
| WEL4 | 34.13 | 0.24 | 1424 | 215 | Ethiopia | Blood | N/A | 1^b^ |
| WEL6 | 32.29 | 0.01 | 4603 | 18 | Ethiopia | Blood | N/A | 1^b^ |
| WEL10 | 28.10 | 0.14 | 68603 | 5988 | Ethiopia | Blood | N/A | 1^b^ |
| WEL11 | 28.99 | 0.01 | 38541 | 354 | Ethiopia | Blood | N/A | 1^b^ |
| WEL12 | 28.27 | 0.16 | 61682 | 6427 | Ethiopia | Blood | N/A | 1^b^ |
| WEL14 | 29.14 | 0.22 | 35286 | 5052 | Ethiopia | Blood | N/A | 1^b^ |
| WEL15 | 27.93 | 0.43 | 78051 | 21473 | Ethiopia | Blood | N/A | 1^b^ |
| WEL16 | 33.91 | 0.40 | 1658 | 419 | Ethiopia | Blood | N/A | 1^b^ |

^1^Ct mean was obtained from duplicate samples within the same run. ^2^Copy number was calculated based on the mean Ct and using the step one software and corresponding batch standards. ^3^All samples were run with the ITS Nested-PCR protocol as per *Scantlebury et al.* 2016 except for the FTA blood samples from Ethiopia. ^a^Cut off Ct values for pus samples were calculated using Receiver Operating Characteristic analysis and set at 27.75. ^b^Receiver Operating Characteristic (ROC) analysis could not be performed on any blood samples as it requires reference test with positive (1)/negative (0) diagnosis; the ITS Nested-PCR could not detect any positive results from blood samples and thus Youden’s index was not appropriate. A mean cut off Ct value of 34.55 was used as an indicator for blood samples.

**Supplementary table 2**. Clinical examination findings.

| **Sample ID** | **Country Sample** | **Clinical Sample Type** | **Species** | **Age** | **Sex** | **Heart rate bpm** | **Respiratory rate rpm** | **Antifungal Treatment** | **Respiratory Signs** | **Ocular Signs** | **Pus Smear yeast observed** | **Temperature** | **Case Severity** |
| --- | --- | --- | --- | --- | --- | --- | --- | --- | --- | --- | --- | --- | --- |
| ANK01P | Gambia | Pus | Horse | 4 | Male | 40 | 36 | no | yes | yes | n/a | 39.4 | Severe |
| DB01P | Gambia | Pus | Horse | 4 | Male | 48 | 40 | no data | no | no | no | 37.8 | Mild |
| DB02P | Gambia | Pus | Donkey | 7 | * | 68 | 24 | no | no | yes | yes | 37.2 | * |
| DB03P | Gambia | Pus | Horse | 3 | Male | 52 |  | no | yes | yes | yes | 38 | Mild |
| KT01P | Gambia | Pus | Horse | 7 | Female | 70 | 20 | yes | no | no | no | 40.4 | Mild |
| KTB01P | Gambia | Pus | Horse | 5 | Male | 56 | 40 | no | yes | yes | yes | 37.7 | Severe |
| KTB02P | Gambia | Pus | Horse | 7 | Male | 60 | 52 | yes | yes | yes | yes | 37.4 | Severe |
| KTB04P | Gambia | Pus | Horse | 7 | Male | 48 | 48 | no | yes | no | yes | 37.9 | Mild |
| KTKL1P | Gambia | Pus | Horse | 5 | Female | 52 | 42 | yes | yes | yes | no | 38.7 | Severe |
| KTKL2P | Gambia | Pus | Donkey | 4 | Male | 60 | 64 | no | yes | no | yes | 37.9 | * |
| KTKL4P | Gambia | Pus | Horse | 3 | * | 44 | 44 | no | yes | no | yes | 37.8 | Mild |
| KTKL5P | Gambia | Pus | Donkey | 7 | Female | 56 | 60 | no | no | no | yes | 38.8 | Severe |
| KTKL6P | Gambia | Pus | Horse | 4 | Male | 36 | 32 | yes | no | no | yes | 38 | * |
| ANK01B | Gambia | Blood | Horse | 4 | Male | 40 | 36 | no | yes | yes | n/a | 39.4 | Severe |
| DB01B | Gambia | Blood | Horse | 4 | Male | 48 | 40 | no data | no | no | no | 37.8 | Mild |
| DB02B | Gambia | Blood | Donkey | 7 | * | 68 | 24 | no | no | yes | yes | 37.2 | * |
| KT01B | Gambia | Blood | Horse | 7 | Female | 70 | 20 | yes | no | no | no | 40.4 | Mild |
| KTB01B | Gambia | Blood | Horse | 5 | Male | 56 | 40 | no | yes | yes | yes | 37.7 | Severe |
| KTB02B | Gambia | Blood | Horse | 7 | Male | 60 | 52 | yes | yes | yes | yes | 37.4 | Severe |
| KTB03B | Gambia | Blood | Horse | 3 | Male | 52 | 48 | no | yes | yes | no | 38.1 | Severe |
| KTB04B | Gambia | Blood | Horse | 7 | Male | 48 | 48 | no | yes | no | yes | 37.9 | Mild |
| KTB05B | Gambia | Blood | Horse | 2 | Female | 52 | 52 | no data | no | no | no | n/a | Mild |
| KTB06B | Gambia | Blood | Horse | 3 | Female | 48 | 56 | no | no | yes | no | 37.3 | Mild |
| KTKL1B | Gambia | Blood | Horse | 5 | Female | 52 | 42 | yes | yes | yes | no | 38.7 | Severe |
| KTKL2B | Gambia | Blood | Donkey | 4 | Male | 60 | 64 | no | yes | no | yes | 37.9 | * |
| KTKL3B | Gambia | Blood | Horse | 9 | Male | 40 | 48 | yes | no | no | no | 37.8 | Mild |
| KTKL4B | Gambia | Blood | Horse | 3 | * | 44 | 44 | no | yes | no | yes | 37.8 | Mild |
| KTKL5B | Gambia | Blood | Donkey | 7 | Female | 56 | 60 | no | no | no | yes | 38.8 | Severe |
| MJK01B | Gambia | Blood | Horse | 10 | Female | 56 | 52 | yes | no | no | no | 38.5 | Mild |
| MJK02B | Gambia | Blood | Horse | 4 | Male | 44 | 32 | no | no | no | no | 38.5 | Mild |
| SNJ01B | Gambia | Blood | Horse | 7 | Male | 36 | 40 | yes | yes | yes | no | 37.6 | Mild |
| SNJ02B | Gambia | Blood | Horse | 6 | Male | 40 | 44 | no | no | no | no | 37.6 | Mild |
| DE1 | Ethiopia | Blood | Horse | 12 | Male | 52 | 36 | n/a | n/a | n/a | n/a | 36.8 | no clinical signs |
| DE3 | Ethiopia | Blood | Horse | 13 | Male | 52 | 52 | n/a | n/a | n/a | n/a | 38.8 | no clinical signs |
| DE6 | Ethiopia | Blood | Horse | 3.5 | Male | 44 | 36 | n/a | n/a | n/a | n/a | 36.3 | no clinical signs |
| SHA6 | Ethiopia | Blood | Horse | 15 | Male | 64 | 60 | n/a | n/a | n/a | n/a | 38.8 | no clinical signs |
| SHA12 | Ethiopia | Blood | Horse | 12 | Male | 44 | 56 | n/a | n/a | n/a | n/a | 37 | no clinical signs |
| WEL2 | Ethiopia | Blood | Horse | 15 | Male | 40 | 44 | n/a | n/a | n/a | n/a | 36.4 | no clinical signs |
| WEL3 | Ethiopia | Blood | Horse | 15 | Male | 44 | 40 | n/a | n/a | n/a | n/a | 37.1 | no clinical signs |
| WEL4 | Ethiopia | Blood | Horse | 15 | Male | 48 | 48 | n/a | n/a | n/a | n/a | 37.3 | no clinical signs |
| WEL6 | Ethiopia | Blood | Horse | 15 | Male | 48 | 64 | n/a | n/a | n/a | n/a | 37.2 | no clinical signs |
| WEL10 | Ethiopia | Blood | Horse | 9 | Male | 44 | 40 | n/a | n/a | n/a | n/a | 37.6 | no clinical signs |
| WEL11 | Ethiopia | Blood | Horse | 15 | Male | 40 | 32 | n/a | n/a | n/a | n/a | 36.4 | no clinical signs |
| WEL12 | Ethiopia | Blood | Horse | 13 | Male | 52 | 32 | n/a | n/a | n/a | n/a | 37.1 | no clinical signs |
| WEL14 | Ethiopia | Blood | Horse | 9 | Male | 40 | 28 | n/a | n/a | n/a | n/a | 36.6 | no clinical signs |
| WEL15 | Ethiopia | Blood | Horse | 7 | Male | 44 | 40 | n/a | n/a | n/a | n/a | 37 | no clinical signs |
| WEL16 | Ethiopia | Blood | Horse | 15 | Male | 56 | 100 | n/a | n/a | n/a | n/a | 39.2 | no clinical signs |

*indicates not recorded / unknown, n/a indicates not available

**Supplementary table 3**. Differential cell count data from blood samples from 18 horses and 3 donkeys in Gambia, and 15 horses in Ethiopia.

| **Sample ID** | **Origin** | **Species** | **PCV** | **Bands** | **Segmented** | **Neutrophils** | **Basophils** | **Eosinophils** | **Monocytes** | **Lymphocytes** |
| --- | --- | --- | --- | --- | --- | --- | --- | --- | --- | --- |
| DB01B | Gambia | Horse | 45 | 5 | 26 | 31 | 0 | 2 | 27 | 40 |
| DB02B | Gambia | Donkey | * | * | * | * | * | * | * | * |
| DB03B | Gambia | Horse | 35 | * | * | * | * | * | * | * |
| KT01B | Gambia | Horse | 14 | * | * | * | * | * | * | * |
| KTB01B | Gambia | Horse | 28 | 12 | 14 | 26 | 9 | 5 | 10 | 50 |
| KTB02B | Gambia | Horse | 32 | 12 | 34 | 46 | 3 | 1 | 19 | 32 |
| KTB03B | Gambia | Horse | * | 9 | 26 | 35 | 5 | 6 | 30 | 24 |
| KTB04B | Gambia | Horse | 35 | 2 | 37 | 39 | 1 | 2 | 56 | 1 |
| KTB05B | Gambia | Horse | 24 | 2 | 46 | 48 | 1 | 1 | 27 | 24 |
| KTB06B | Gambia | Horse | * | * | * | * | * | * | * | * |
| ANK01B | Gambia | Horse | 35 | 0 | 22 | 22 | 4 | 0 | 56 | 18 |
| KTKL1B | Gambia | Horse | 38 | 6 | 34 | 40 | 3 | 0 | 47 | 8 |
| KTKL2B | Gambia | Donkey | 26 | * | * | * | * | * | * | * |
| KTKL3B | Gambia | Horse | 34 | 1 | 19 | 20 | 0 | 3 | 53 | 24 |
| KTKL4B | Gambia | Horse | 43 | 0 | 60 | 60 | 1 | 0 | 28 | 11 |
| KTKL5B | Gambia | Donkey | 41 | 1 | 23 | 24 | 3 | 5 | 45 | 22 |
| KTKL6B | Gambia | Horse | 30 | 0 | 42 | 42 | 0 | 5 | 42 | 11 |
| MJK01B | Gambia | Horse | 46 | 2 | 20 | 22 | 2 | 6 | 64 | 6 |
| MJK02B | Gambia | Horse | 40 | * | * | * | * | * | * | * |
| SNJ01B | Gambia | Horse | 38 | 6 | 30 | 36 | 0 | 10 | 34 | 20 |
| SNJ02B | Gambia | Horse | 36 | 3 | 8 | 11 | 2 | 7 | 60 | 20 |
| DE1 | Ethiopia | Horse | 44 | * | * | * | * | * | * | * |
| DE3 | Ethiopia | Horse | 32 | * | * | * | * | * | * | * |
| DE6 | Ethiopia | Horse | 30 | * | * | * | * | * | * | * |
| SHA6 | Ethiopia | Horse | 33 | 0 | 32 | * | 0 | 2 | 2 | 62 |
| SHA12 | Ethiopia | Horse | 34 | 0 | 34 | * | 0 | 4 | 2 | 60 |
| WEL2 | Ethiopia | Horse | 20 | 0 | 50 | * | 0 | 8 | 2 | 40 |
| WEL3 | Ethiopia | Horse | 30 | 0 | 31 | * | 0 | 8 | 1 | 60 |
| WEL4 | Ethiopia | Horse | 24 | 0 | 58 | * | 0 | 2 | 4 | 36 |
| WEL6 | Ethiopia | Horse | 30 | 0 | 36 | * | 0 | 10 | 4 | 50 |
| WEL10 | Ethiopia | Horse | 32 | 0 | 28 | * | 0 | 12 | 4 | 56 |
| WEL11 | Ethiopia | Horse | 30 | 0 | 50 | * | 0 | 4 | 2 | 44 |
| WEL12 | Ethiopia | Horse | 30 | 2 | 34 | * | 0 | 6 | 2 | 56 |
| WEL14 | Ethiopia | Horse | 32 | 0 | 46 | * | 0 | 4 | 0 | 50 |
| WEL15 | Ethiopia | Horse | 28 | 0 | 30 | * | 0 | 12 | 0 | 58 |
| WEL16 | Ethiopia | Horse | 26 | 0 | 54 | * | 0 | 4 | 2 | 20 |

*indicates not tested, this may have been due to issues with sample volume.
